# Emotional non‐acceptance mediates the relationship between insecure attachment and specific psychopathology in women with eating disorders

**DOI:** 10.1192/j.eurpsy.2024.1161

**Published:** 2024-08-27

**Authors:** S. Donato, N. Attianese, M. Battipaglia, R. Ceres, R. Cerra, A. M. Monteleone, P. Monteleone, G. Cascino

**Affiliations:** ^1^University of Campania “L.Vanvitelli”, Naples; ^2^Universiy of Salerno, Salerno, Italy

## Abstract

**Introduction:**

Insecure attachment is considered a general risk factor for eating disorders (ED). Emotion dysregulation has been proposed as one of the possible mechanisms by which attachment insecurity may affect ED psychopathology.

**Objectives:**

Aim of the present study was to investigate whether difficulties in acceptance of emotions or emotional clarity may mediate the connection between insecure attachment and ED psychopathology.

**Methods:**

One hundred and twenty patients participated and completed the Italian version of Eating Disorder Inventory‐2 (EDI-2), Experience in Close Relationship questionnaire (ECR) and Difficulties in Emotion Regulation Scale (DERS). A mediator path model was performed, in which insecure attachment dimensions were set as independent variables, ED specific psychopathology measures as dependent variables, and non‐acceptance of emotion and lack of emotional clarity as mediators.

**Results:**

The association between both attachment avoidance and anxiety and ED specific symptoms was mediated by emotional non‐acceptance, but not by emotional clarity.

**Conclusions:**

This study showed the importance to address emotion regulation in individuals with ED, focussing on improving emotional acceptance. Exploring early developmental processes which lead to non‐acceptance of emotions could improve this psychological trait in people with ED.

**Disclosure of Interest:**

None Declared

